# Effect of *Nauclea subdita* (Korth.) Steud. leaf extract on hematological and histopathological changes in liver and kidney of striped catfish infected by *Aeromonas hydrophila*

**DOI:** 10.14202/vetworld.2020.47-53

**Published:** 2020-01-09

**Authors:** Siti Aisiah, Arief Prajitno, Maftuch Maftuch, Ating Yuniarti

**Affiliations:** 1 Doctoral Program of Fisheries and Marine Sciences, Faculty of Fisheries and Marine Sciences, Brawijaya University, Malang, East Java 65145, Indonesia; 2 Department of Aquaculture, Faculty of Fisheries and Marine Sciences, Lambung Mangkurat University, Banjarbaru, South Kalimantan 70714, Indonesia; 3 Department of Aquaculture, Faculty of Fisheries and Marine Sciences, Brawijaya University, Malang, East Java 65145, Indonesia

**Keywords:** *Aeromonas hydrophila*, hematology, histopathology, *Nauclea subdita*

## Abstract

**Aim:**

The present study was conducted to investigate the efficacy of different doses of Bangkal (*Nauclea subdita*) leaf extract on hematological and histological changes in kidney and liver of catfish (*Pangasius hypophthalmus*) infected by *Aeromonas hydrophila*.

**Materials and Methods:**

Catfish were experimentally infected with *A. hydrophila* at a dose of 10^8^ cells/mL through intraperitoneal injection, and the hematological and histological changes in the kidney and liver of catfish against the pathogen were observed.

**Results:**

Not all concentrations of *N. subdita* caused a toxic effect in striped catfish. The clinical symptoms of catfish after infection with *A. hydrophila* and treatment with *N. subdita* leaf extract included morphological and behavioral changes. *N. subdita* leaf extract reduced mortality caused by *A. hydrophila*. Treatment with *N. subdita* leaf extract was effective in reducing the inflammation by decreasing the activity of neutrophils, monocytes, and lymphocytes. The Hb and Ht levels of catfish significantly decreased after exposure to 10^8^ cells/mL of *A. hydrophila* and increased significantly after *N. subdita* treatment. Necrosis percentages in the kidney and liver also decrease after *N. subdita* treatment.

**Conclusion:**

The results indicate that *N. subdita* leaf extract stimulates the immunity and increases the resistance of catfish to *A. hydrophila. N. subdita* leaf extract may be used as a potential source for future drug development and food applications.

## Introduction

Fish farming in Indonesia, especially in South Kalimantan, is growing rapidly as a result of the increasing demand for fish. Catfish (*Pangasius hypophthalmus*) are a popular freshwater fish that are easily cultivated under marginal conditions. Intensive aquaculture with high stocking capacity can cause a decrease in environmental quality, making fish susceptible to diseases [1]. Fish experiencing diseases and stress due to environmental degradation die quickly by a lack of oxygen. Environmental degradation also allows the development and growth of pathogenic organisms [2-4]. Infectious diseases caused by bacteria are a severe problem in fish farming. Motile Aeromonas septicemia (MAS) or red spot disease is an infectious disease caused by *Aeromonas hydrophila* and often occurs in carp, catfish, and tilapia. MAS attack on a fish stock is often catastrophic as it can kill fish seeds with mortality rates reaching 80-100% within 1-2 weeks [5]. The pathogen also spreads rapidly in high stocking densities [6]. The epidemic disease caused by *A. hydrophila* often occurs during the transition from the dry season to the rainy season.

A typical treatment to control MAS is the use of chemicals or antibiotics, but this creates bacterial resistance to antibiotics if used continuously. Another negative impact is the accumulation of these antibiotics in tissues, especially bone tissue, which poses health risks to consumers [7]. Drug and synthetic antibiotic residues accumulate in fish meat, kill non-target organisms, result in antibiotic drug resistance, affect fish growth and reproductive ability, and cause environmental pollution. Therefore, an alternative such as the bioactive compounds from plants with natural antibacterial properties is needed to control the disease in a safe and environmental-friendly manner.

*Nauclea subdita* (Korth.) Steud. is a semiaquatic plant that grows in a wetland, Kalimantan, Indonesia [8]. The active compounds such as phenolics, saponins, tannins, and alkaloids in the plant potentially eliminate pathogenic bacteria [9]. The inhibition zone created by the skin and leaves is larger than for the other parts of the plant. However, these studies are limited in simple extraction, thus the obtained results do not significantly contribute to both knowledge and society.

This study aimed to analyze the effectiveness of the bioactive compounds of Bangkal (*N. subdita)* (Korth.) Steud. as a natural bactericide for controlling *A. hydrophila* in catfish aquaculture.

## Materials and Methods

### Ethical approval

This study was approved by the Animal Care and Use Committee, Brawijaya University, Malang, Indonesia (approval no. 940-KEP-UB).

### Research design

A total of 15 adult catfish (*P. hypophthalmus*) were used in this study. Healthy catfish seeds (length 10-12 cm) were procured from the Mojokerto Fish Seed Center. Before the experimental tests, the fish were acclimatized for 7-10 days in a maintenance media (15 L capacity) under laboratory conditions. A completely randomized design with five treatment groups was used in this study, and each treatment was repeated 3 times:

K=Healthy fish without *A. hydrophila* infection

A=Fish with *A. hydrophila* infection without treatment

B=Fish with *A. hydrophila* infection and an *N. subdita* leaf extract dose of 50 mg/L

C=Fish with *A. hydrophila* infection and an *N. subdita* leaf extract dose of 100 mg/L

D=Fish with *A. hydrophila* infection and an *N. subdita* leaf extract dose of 150 mg/L.

### Toxicity study of *N. subdita* leaf extract

Five fish seeds were placed into five research containers with 10 L of water. Then, *N. subdita* leaf extract was added into each container with the following concentration: 62.5 mg/L, 125 mg/L, 250 mg/L, 500 mg/L, and 1000 mg/L. For each concentration, three replicates were maintained. The mortality of fish was observed every 12 h for 96 h, and the concentration resulting in 50% mortality was determined as the lethal dose (LD_50_).

### Pathogenicity study of *A. hydrophila*

For the pathogenicity test, five catfish were introduced into five research containers with 10 L water, and *A. hydrophila* was added into each container with the following densities: 10^6^ cells/mL, 10^7^ cells/mL, 10^8^ cells/mL, 10^9^ cells/mL, and 10^10^ cells/mL. The survival rate of the fish was recorded after 24 h, and LD_50_ was determined.

### Lethal toxicity test and *N. subdita* treatment

Ten fish were placed into 10 research containers, and a suspension of *A. hydrophila* was added to five experimental containers. The symptoms of infection were observed, and after 24-36 h, *N. subdita* leaf extract was added in different treatment doses of 50 mg/L, 100 mg/L, and 150 mg/L [10]. The observations and measurements were carried out every 24 h. The mortality rate of the fish was determined at the end of the study. Water quality parameters including temperature, pH, and dissolved oxygen were recorded at the beginning and end of the study.

### Blood sample collection

Blood was collected from the control and treated groups using a microhematocrit. The collected blood was added to a 1 mL tube, which already included an anticoagulant, and Hb, red blood cells, and white blood cells were measured. The remaining blood samples were centrifuged at 12,000 rpm for 5 min.

### Hematological analysis

RBC and WBC were counted by a hemocytometer. The Hb concentration was measured using the Bijanti method (Bijanti, 2005). Erythrocytes were calculated according to the standard formula:


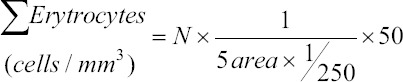



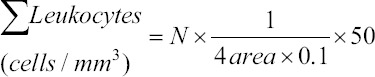


### Leukocytes differentiation

The differentiation of leukocytes was analyzed in blood smears stained with 10% Giemsa and observed under a microscope with 100×. The type of leukocytes was determined and calculated until a total of 100 cells were counted.

### Histopathological analysis

At the end of the experiment, three fish from each group were sampled after 96 h of exposure for histological analysis. The treated fish were killed and dissected to collect the kidney and liver. The organs were fixed in 10% formalin and prepared for Hematoxylin-Eosin (H&E) staining [11]. The stained slide was observed under a light microscope at 400×.

### Statistical analysis

The data were statistically analyzed at p<0.05 and significance was calculated by an analysis of variance test using SPSS for Windows.

## Results and Discussion

### Toxicity test of *N. subdita* leaf extract

**Figure-1:**
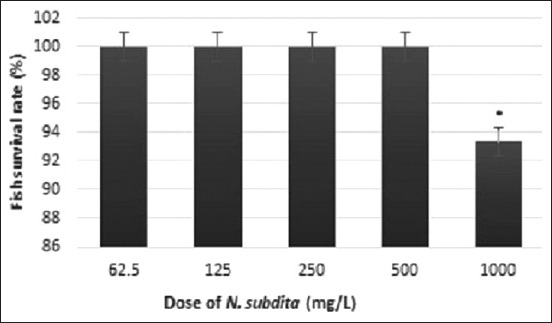
Average of survival rate of catfish with *Nauclea subdita* leaf extract treatment.

The toxicity test of *N. subdita* to *P. hypophthalmus* did not result in dead fish in any of the experimental groups. The survival rate of fish was higher than 92% under the administration of *N. subdita* at any dose (Figure-1). It was concluded that *N. subdita* had no toxic effect on striped catfish at any dose concentrations.

### Pathogenicity test of *A. hydrophila*

The pathogenicity of *A. hydrophila* to catfish was determined by 100% mortality at a dose of 1 × 10^10^ cells/mL after 48 h and 1 × 10^9^ cells/mL after 96 h. It was concluded that catfish were susceptible to *A. hydrophila* at doses of 1 × 10^9^ cells/mL and 1 × 10^10^ cells/mL, resulting in redness of skin and slow movements (Table-1).

**Table-1:** The pathogenicity test of *A. hydrophila* to catfish (*P.* *hypophthalmus*).

Density	Number of fish	Observation					

		0 h	12 h	24 h	48 h	72 h	96 h
10^6^	5	Normal	Normal	Decreased appetite	Mouth and tail redness	Flickertail	One fish dead
10^7^	5	Normal	Normal	Decreased appetite	Flickertail	Two fishes dead	**-**
10^8^	5	Normal	Not active	Flickertail	One fish dead	Two fishes dead	Three fishes dead
10^9^	5	Normal	Not active	Two fishes dead	Three fishes dead	Four fishes dead	Five fishes dead
10^10^	5	Normal	Skin redness	Four fishes dead	Five fishes dead	**-**	**-**
Control	5	Normal	Normal	Normal	Normal	Normal	Normal

*A. hydrophila=Aeromonas hydrophila*, *P.* *hypophthalmus=Pangasius* *hypophthalmus*

### Clinical signs after *N. subdita* treatment

A dose of 1 × 10^8^ cells/mL of *A. hydrophila* was used for the survival test to infect the striped catfish for 96 h. After 24 h of treatment with *N. subdita*, clinical signs appeared in the treated fish (Table-2). The results were consistent with those of Abdelhamed *et al*. [12], where clinical signs were present 24 h after *A. hydrophila* infection until few days after infection, depending on the size and immunity of the fish. In another study, the clinical signs appeared 6 h after infection with *A. hydrophila* [13].

**Table-2:** The clinical signs of catfish after *A. hydrophila* infection and *N. subdita* treatment.

Time	Symptoms	

*A. hydrophila*-infected fish with *N. subdita* treatment	*A. hydrophila*-infected fish
12 h	Disturbed balance, swimming abnormalities, swimming slowly, disturbed eating response	Disturbed balance, swimming abnormalities, swimming slowly, disturbed eating response
24 h	No eating response, sluggish movement	No eating response, sluggish movement
36 h	No eating response, sluggish movement, some fish fin start to swell and jagged	No eating response, sluggish movement, some fish fin are jagged
2 days	Jagged fin look more clearly, suffering from dropsy	Three fish with inflammation and jagged fin were dead, three dropsy fish were dead and some of fish still dropsy, refusing food and sluggish movement
3 days	Inflammation and jagged fin, dropsy were observed, but fish start to eat and active movement	Two inflammation fish were dead and three dropsy fish were dead, the low of eating response
4 days	Inflammation starts to diminish, dropsy cured, swimming in a normal and active	Three hemorrhagic fish were dead, two dropsy fish died, no response to feeding and slow movement
5 days	Inflammation gets smaller, normal swimming with active movement, and response to feed is good	The slow movement, one hemorrhagic fish dies
6 days	No more inflammation, active swimming, good response to feed	Not an active movement, no eating response, and fish still hemorrhagic
7 days	No more inflammation, active swimming, good response to feed	Not an active movement, no eating response, and fish still hemorrhagic

*A. hydrophila=Aeromonas hydrophila*, *N. subdita*=*Nauclea subdita*

Clinical symptoms after infection included empty intestines, changes in body color, erroneous swimming behavior, reddish-colored tails, and jagged caudal fin [14] (Table-2). The catfish showed morphological and behavioral changes after infection with *A. hydrophila* and treatment with *N. subdita* leaf extract. The symptoms appeared 24 h after infection with *A. hydrophila* by the immersion method.

The results showed that *N. subdita* leaf extract could reduce the mortality caused by the *A. hydrophila-*induced MAS disease. The active compounds in *N. subdita* leaves are flavonoids, saponins, tannins, and triterpenoids based on phytochemical tests. According to the previous studies, these compounds have antibacterial properties inhibiting bacterial growth. No dead fish were found in this study after *N. subdita* leaf extract treatment, which might be a result of the phenolic and flavonoid compounds acting as an antioxidant. The phenolic compounds contribute to the oxidative mechanism by degrading reactive oxygen species. Antioxidants decrease oxidative stress and may be used as a supplement to prevent the disease [15].

### Leukocyte differentiation

**Figure-2:**
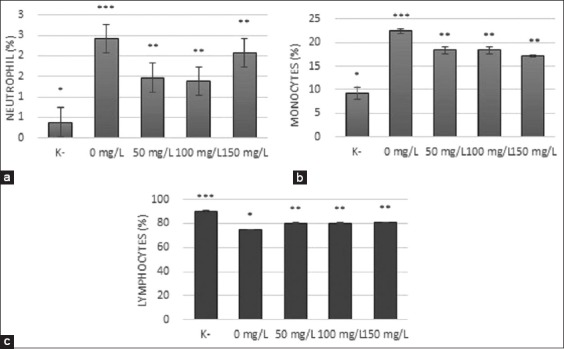
The level of (a) neutrophil, (b) monocytes, and (c) lymphocytes after *Nauclea subdita* leaf extract treatment in *Aeromonas hydrophila*-treated striped catfish. K^−^=Healthy fish; 0 mg/L=Infected fish without treatment; 50 mg/L=Infected fish and administration of 50 mg/L of *N. subdita* leaf extract; 100 mg/L=Infected fish and administration of 100 mg/L of *N. subdita* leaf extract; 150 mg/L: Infected fish and administration of 150 mg/L of *N. subdita* leaf extract.

The results of the study showed that the neutrophil level was significantly increased (2.42%) after *A. hydrophila* infection compared to healthy fish (0.38%) (Figure-2a). After treatment with *N. subdita* leaf extract, the level of neutrophils significantly decreased at all dose concentrations. However, the lowest level of neutrophils was found after an *N. subdita* treatment (1.388%) with a dose of 100 mg/L followed by treatments with a dose of 50 mg/L (1.47%) and 150 mg/L (2.08%). The increase in neutrophils contributed to the antimicrobial activity. Neutrophils in the blood increase after infection and act as the first defense in the body [16]. According to Sahan *et al*. [17], neutrophils play a role in defense against bacterial infections and migrate to blood vessels in the presence of inflammatory stimulation.

The level of monocytes after *N. subdita* treatment was also significantly decreased at all dose concentrations (18.38%, 18.37%, and 17.15%) (p<0.05) compared to infected fish (22.44%) (Figure-2b). The lowest level of monocytes was found at a dose of 100 mg/L. Monocytes are the precursor of macrophages, which are involved in defense against bacterial infection through phagocytic mechanisms [2]. The level of lymphocytes decreased significantly (75.14%) after infection with *A. hydrophil* a compared to the healthy fish (90.30%) by transforming into B-cells and producing antibodies to eliminate the bacteria [18].

Administration of *N. subdita* leaf extract to the infected fish caused an increase in the percentage of lymphocyte at all dose concentrations (80.17%, 80.24%, and 80.78%) (Figure-2). The increase in leukocytes resulted from the activation of neutrophils, monocytes, and lymphocytes to eliminate the bacteria through several mechanisms. This study showed that treatment with *N. subdita* leaf extract was effective in reducing the inflammation by decreasing the activation of neutrophils, monocytes, and lymphocytes.

### Hematological parameters

The Hb and Ht of catfish significantly decreased after exposure to sublethal concentrations of *A. hydrophila* (10^8^ cells/mL) (7.63 mg/dl) compared to healthy fish (9.40 mg/dl) (Table-3). The average Hb level in the experimental fish ranged between 9.30 and 10.33 mg/dl, while the average Hb level in catfish is around 9.40 mg/dl. The Hb value increased significantly (p<0.05) after *N. subdita* leaf extract treatment compared to *A. hydrophila*-infected fish. The increase in Hb was found for all dose concentrations in the *N. subdita* treatment, and the highest level of Hb was found for a dose of 100 mg/L of *N. subdita*.

**Table-3:** The hematological parameter observed after *N. subdita* treatment in *A. hydrophila*-infected catfish.

Hematological parameters	Group treatment				

	Healthy fish	Infected fish	50 mg/L	100 mg/L	150 mg/L
Hb (g/100 mL)	9.40^b^	7.63^a^	9.33^c^	10.33^d^	9.30^c^
Ht (%)	26.67^b^	21.33^a^	24.33^c^	26.33^d^	25.00^c^

a,b,c,d Values in rows denoted with different letter differ significantly at p<0.05. *A. hydrophila=Aeromonas hydrophila*, *N. subdita*=*Nauclea subdita*

There was a decrease in hemoglobin in treated fish due to competition for oxygen between the fish and the pathogen: The level of hemoglobin in fish varies with fish species, blood pH, environmental conditions, and oxygen partial pressure [19]. Hb is involved in the catabolism process to produce energy by binding oxygen. The oxygen-binding ability of blood depends on the level of hemoglobin contained in the erythrocytes. Hidayat *et al*. [20] reported that low Hb levels caused a decrease in the metabolism rate and energy production. Therefore, low Hb levels made some fish move slowly and have no eating response. This clinical sign can be observed 12 h after infection with *A. hydrophila* in catfish (Table-2).

The results showed that after *A. hydrophila* infection, the level of Ht was significantly decreased compared to healthy fish (26.67% vs. 21.33%). A dose of 100 mg/mL *N. subdita* resulted in the highest level of Ht (26.33%) compared to the other dose concentrations (50 mg/mL = 24.33%; 150 mg/mL = 25%) (Table-3). The hematocrit level varies with nutritional factors, age, sex, body size, and spawning period. Hematocrit can be used as a parameter to determine the health status and abnormalities of fish. According to Shen *et al*. [21], the level of Ht in *Teleostei* fish ranges from 20 to 40%. A hematocrit level of <22% will lead to anemia in fish. Anemia has an impact on fish metabolism and growth as the low number of erythrocytes results in reduced nutrient supply to the cells, tissues, and organs [16]. The increase in Ht after *N. subdita* treatment indicated that the health status of the infected fish also increased.

According to Table 4, the blood plasma of healthy fish was usually clear white or clear yellowish. The blood plasma in the treated fish was reddish clear, which indicated the presence of hemolysis caused by *A. hydrophila* infection, as demonstrated by Mastan [22].

**Table-4:** Blood plasma color in fish.

Treatment (mg/L)	Sampling		

	1	2	3
K	Clear white	Clear white	Clear white
0	Clear white	Yellowish clear	Reddish clear
50	Clear white	Yellowish clear	Reddish clear
100	Clear white	Yellowish clear	Yellowish clear
150	Clear white	Yellowish clear	Yellowish clear

### Histopathological changes on liver and kidney

**Figure-3:**
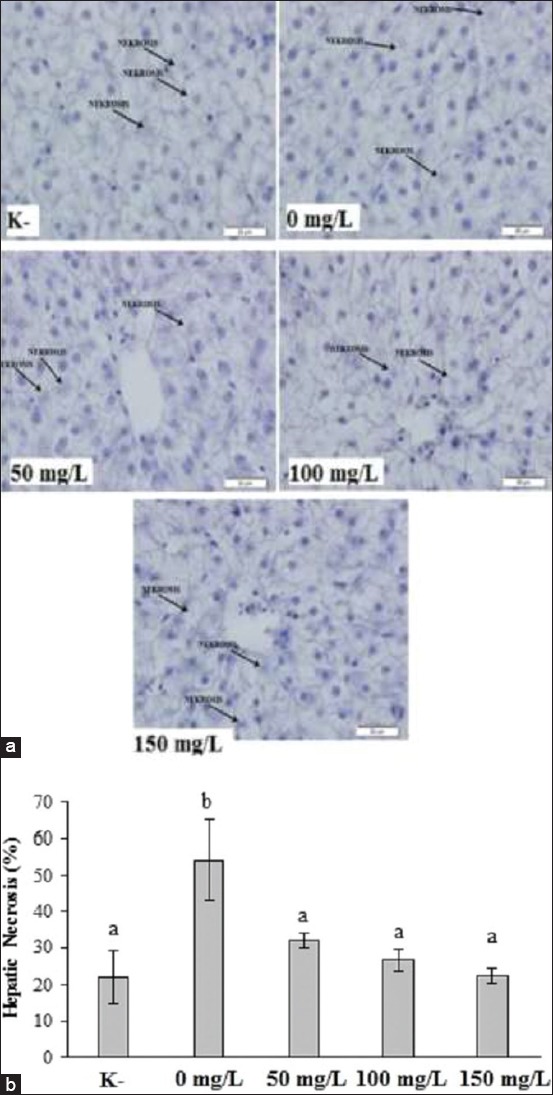
Histopathology overview in healthy striped catfish and striped catfish challenged with *Aeromonas hydrophila* with/without *Nauclea subdita* treatment. (a) Hepatic section of striped catfish at 100× and (b) hepatic necrosis was represented as mean ± standard deviation. The different letter on the chart considered as significantly different for each group at p<0.05 based on the Tukey test as a *post hoc* test. K^-^=Healthy fish; 0 mg/L=Infected fish without treatment; 50 mg/L=Infected fish and administration of 50 mg/L of *N. subdita* leaf extract; 100 mg/L=Infected fish and administration of 100 mg/L of *N. subdita* leaf extract; 150 mg/L=Infected fish and administration of 150 mg/L of *N. subdita* leaf extract).

The results suggest that *A. hydrophila* infection increased hepatic necrosis significantly (p<0.05) in infected catfish compared to healthy fish. *N. subdita* leaf extract treatment decreases the hepatic necrosis significantly (p<0.05) compared to infected fish (Figure-3).

Hepatic tissue infected with *A. hydrophila* showed necrosis and swelling of hepatic cells, characterized by the presence of vacuoles. Cell swelling occurs due to an unbalance in electrolytes between the inner and outer cells [23]. The cells pumping fluid outward cause an inward movement of extracellular fluid; meanwhile, the cells lose their ability to pump sodium ions. As a result, the cells will lose their integrity, leading to cell death (necrosis). Cell swelling will diminish if the bacterial infection disappears. However, the cell cannot tolerate the damage caused by a prolonged infection and will undergo necrosis [24].

**Figure-4:**
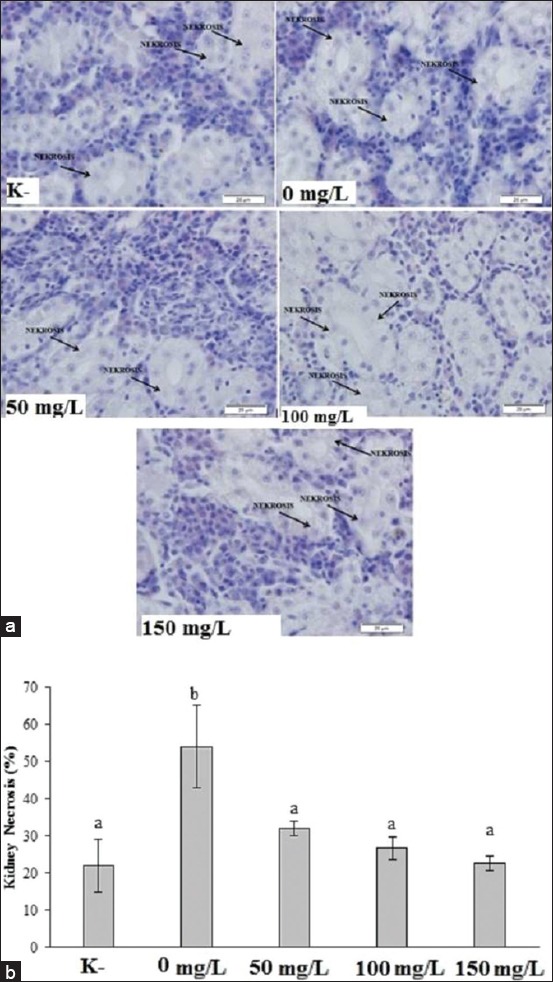
Histopathology overview in healthy striped catfish and striped catfish challenged with *Aeromonas hydrophila* with/without *Nauclea subdita* treatment. (a) Kidney section of striped catfish at 100× and (b) kidney necrosis was represented as mean ± standard deviation. The different letter on the chart considered as significantly different for each group at p<0.05 based on the Tukey test as a *post hoc* test. K^-^=Healthy fish; 0 mg/L=Infected fish without treatment; 50 mg/L=Infected fish and administration of 50 mg/L of *N. subdita* leaf extract; 100 mg/L=Infected fish and administration of 100 mg/L of *N. subdita* leaf extract; 150 mg/L=Infected fish and administration of 150 mg/L of *N. subdita* leaf extract

*A. hydrophila* infection increased kidney necrosis significantly (p<0.05) in *Pangasius* fish compared to healthy fish. *N. subdita* leaf extract treatment decreases the kidney necrosis significantly (p<0.05) compared to infected fish (Figure-4).

The kidney necrosis is caused by swollen epithelial cells in the renal tubules, triggering glomerulus proliferation followed by lysis in the Bowman capsule. Kidneys are the excretory organs and thus susceptible to pathogens. The kidneys have two main functions: Excreting most of the final metabolic products and regulating the body fluid concentration [25].

*N. subdita* contains secondary metabolites such as tannins, alkaloids, and phenolic compounds, which act as a natural bactericide [26]. The leaves, bark, and root of *Nauclea latifolia, N. subdita*, and *Nauclea officinalis* are also known to contain alkaloids, flavonoids, and tannins [27-29]. Some phytochemicals such as tannins, alkaloids, and flavonoids are proven to have antibacterial properties in fish [30].

The methanolic extract of the bark and leaves of *Nauclea obversifolia* demonstrated a higher antibacterial effect, suggesting that fractionation increases the antibacterial activity of bark and skin root. The ethyl acetate fraction was most effective in inhibiting bacterial growth [31]. The extract of the *N. latifolia* root is more effective than the bark and leaf extract [32]. Plant extract contains active compounds that mimic synthetic drugs in *in vitro* studies [33]. Tannins perform as a bacteriostatic and a bactericide against pathogenic bacteria, including *A*. *hydrophila, Escherichia coli, Listeria, Pseudomonas, Salmonella*, *Staphylococcus*, and *Streptococcus* [34].

Phytochemicals such as polyphenol are also expected to have an inhibitory effect on bacteria. Tannins have toxicity and inhibit some enzyme in microorganisms [35]. The *N. latifolia* extract contains polyphenol, an antioxidant and antibacterial compound fighting pathogens, such as *Staphylococcus aureus, Streptococcus pyogenes, E. coli*, and *Candida* [36].

## Conclusion

The present study demonstrated that *N. subdita* leaf extract did not have a toxic effect on stripped catfish at 50 mg/L, 100 mg/L, and 150 mg/L dose concentrations. Furthermore, *N. subdita* extract reduced the mortality and inflammation, increased the hemoglobin and hematocrit values, and improved kidney and liver necrosis after infection with *A. hydrophil*a.

## Authors’ Contributions

SA contributed to the conception, research design, and designed manuscripts. AP did data acquisition. MM did analysis and/or interpretation of data. AY improved the manuscript. All authors read and approved the final manuscript.
